# Editorial: Rising stars in cancer metabolism 2022

**DOI:** 10.3389/fonc.2023.1223630

**Published:** 2023-05-31

**Authors:** Jose Luis Izquierdo-Garcia, Domenica Scumaci

**Affiliations:** ^1^ Biomedical Imaging Group, Instituto Pluridisciplinar, Complutense University of Madrid, Madrid, Spain; ^2^ Department of Chemistry in Pharmaceutical Sciences, Pharmacy School, Complutense University of Madrid, Madrid, Spain; ^3^ Centro de Investigación Biomédica en Red de Enfermedades Respiratorias (CIBERES), Madrid, Spain; ^4^ Department of Experimental and Clinical Medicine, Magna Græcia University of Catanzaro, Catanzaro, Italy; ^5^ Research Center on Advanced Biochemistry and Molecular Biology, Magna Græcia University of Catanzaro, Catanzaro, Italy

**Keywords:** metabolic reprogramming, metabolic symbiosis, glutamine metabolism, metabolism and redox homeostasis, metabolism and chemoresistance, personalized medicine

Recognizing and supporting the next generation of leaders in oncology is crucial for ensuring that we continue to drive innovation and make progress in the fight against cancer. This editorial collection focuses on early-career researchers who have already established themselves as internationally recognized experts in Cancer Metabolism, a rapidly growing area of research that studies metabolic changes in cancer cells (1). By understanding these processes, researchers can develop innovative strategies for diagnosing and treating cancer (2). The collection presents cutting-edge research conducted by future leaders of the discipline, with real-world applications to pressing challenges in cancer research. The study of cancer metabolism has the potential to transform cancer treatment and improve patient outcomes.

During carcinogenesis, cells undergo dramatic metabolic rewiring, acquiring the ability to survive in hard condition. Cancer is a dynamic disease and therefore tumor microenvironment results in heterogeneous cells population characterized by peculiar molecular signatures including those involving metabolism. In this perspective, the article of Amemiya and Yamaguchi aims to address the notion of a metabolic symbiosis between cancer and tumor microenvironment. The authors propose that the co-culture of cancer cells and CAFs might represent a smart model to investigate “real-time” the metabolic oscillations at the single-cell level unveiling the metabolic heterogeneities that surround cell symbiosis. In this scenario, being metabolic cross-talk a driver of invasion, resistance to chemotherapy and malignancies grade, it might represent a strategic target to implement personalized cancer treatments.

On cancer heterogeneity, is also focused the paper of Yi et al. Here the authors integrate omics approaches to profile multiple samples of endometrial tissues disclosing that amino acid and nucleotide metabolism have a crucial role in endometrial cancer (EC). The major strength of this work was the use of multiple samples that allowed the identification of a subset of putative metabolic biomarkers recognizable by using minimally invasive procedures.

Colorectal cancer (CRC) is a prevalent malignancy worldwide and metastasis to the liver and lung is common. Metabolic reprogramming has been implicated in CRC progression and metastasis. In this Editorial, two recent studies shed light on the metabolic pathways involved in CRC and the potential therapeutic targets that may emerge from this research. Montero-Calle et al. investigated the metabolic and functional differences between two CRC cell lines with different metastatic organotropisms. The study identified several altered lipid metabolism-related targets, including LDLR, CD36, FABP4, SCD, AGPAT1, and FASN, which were associated with the prognosis of CRC patients. The study also found that CD36 was associated with lung metastatic tropism of CRC cells, validating the *in vivo* relevance of the findings. These results suggest specific metabolic adaptations for invasive cancer cells, which could serve as potential therapeutic targets.

In a second study, Hartal-Banishay et al. investigated the role of sterol regulatory element-binding proteins (SREBPs) in CRC proliferation. The study found that membrane-bound transcription factor protease 1 (MBTPS1) is critical for the proliferation of CRC cells. Inhibition of MBTPS1 activity decreased SREBP levels and cell proliferation in CRC-derived cell lines, while CRISPR/Cas9 KO of the MBTPS1 gene resulted in severely attenuated proliferation and downregulation of several energy metabolism pathways. These findings suggest that MBTPS1 plays a critical role in regulating colon cancer proliferation primarily through SREBP-associated lipid metabolism and may serve as a possible therapeutic target in CRC. Taken together, these studies highlight the importance of understanding the metabolic pathways involved in CRC progression and metastasis. The identification of specific metabolic targets, such as CD36 and MBTPS1, may lead to the development of novel therapeutic strategies for CRC.

Recently, several studies pointed out the role of Glutamine metabolism in fueling energy metabolism as well as in maintaining oxidative homeostasis. In this perspective, the review of Gong et al. emphasizes the molecular mechanisms that relate glutamine metabolism and oxidative homeostasis. Here the authors, underling the importance of redox homeostasis relying on glutamine metabolism in cancer cells, endorse the development and improvement of strategies aiming to interfere with this relationship.

A further emergent metabolic pathway that seems to have a key role in redox homeostasis is the glycolytic branch named Hexosamine Biosynthetic Pathway (HBP). That is the main theme of the work of Zerbato et al. that propose a strategy to improve cancer therapy in Pancreatic ductal adenocarcinoma. Authors point out that the use of a novel Phosphoglucomutase 3 (PGM3) enzyme inhibitor, named FR054, sensitizes cancer cells to Erastin treatment by altering the Unfolded Protein Response (UPR).

Wang et al. explored the correlation between ATP-binding cassette (ABC) transporters and immunomodulation in thyroid carcinoma (TC), using data from The Cancer Genome Atlas (TCGA) database. Five hallmark ABC transporters were identified as prognostic factors for TC and were associated with the relapse-free survival rates of patients. These transporters were found to modulate various aspects of immune cell infiltration, and their expression was affected by certain chemicals. Understanding the role of these transporters may lead to potential prognostic and immunotherapeutic strategies for TC.

Understanding metabolic changes during cancer treatments is crucial. Astrocytomas are the most common type of brain tumors, and radiotherapy (RT) is commonly used to treat them. However, monitoring treatment response using magnetic resonance imaging (MRI) only captures structural changes, while molecular changes may occur without visible structural changes. Ruiz-Rodado et al. used liquid chromatography mass spectrometry (LC/MS) and nuclear magnetic resonance (NMR) to identify plasma and tissue metabolic biomarkers of treatment response in a mouse model of astrocytoma undergoing RT. The results showed metabolic changes in mice that underwent RT, and identified fumarate as the best discriminatory feature in plasma. The study suggests these biomarkers could be validated in the clinic to improve the assessment of brain tumor patients throughout radiotherapy.

Lastly, an interesting review of Zhou et al. pointed out the role of human microbiome in maintaining metabolites homeostasis underlying a promising field of investigation. The authors critically discuss recent literature on tumor microbiome metabolism suggesting it as a novel player in the homeostasis of tumor microenvironment metabolites opening an innovative perspective to understand cancer progression and develop novel therapeutic opportunities.

## Author contributions

All authors listed have made a substantial, direct, and intellectual contribution to the work, and approved it for publication.
